# Lipid profiles and nutritional dynamics of long‐distance hiking: A longitudinal study on the Colorado Trail

**DOI:** 10.1113/EP093118

**Published:** 2025-12-28

**Authors:** Kiaya Johnston, Stephen Selinsky, Benjamin Langworthy, Daniel Craighead

**Affiliations:** ^1^ Department of Medicine University of Minnesota Minneapolis Minnesota USA; ^2^ Division of Biostatistics & Health Data Science Minnesota School of Public Health Minneapolis Minnesota USA; ^3^ School of Kinesiology University of Minnesota Minneapolis Minnesota USA

**Keywords:** cholesterol, metabolic health, thru‐hiking

## Abstract

Current literature on the metabolic effects of long‐distance hiking is limited to case studies with discrepant findings, and no prior studies have examined the role of diet in shaping these outcomes. In this study, we investigated changes in lipid profiles and dietary factors among 12 participants who completed the Colorado Trail. Blood lipid measures [low‐density lipoprotein cholesterol (LDL‐C), high‐density lipoprotein cholesterol, total cholesterol and triglycerides] were obtained pre‐ and post‐trail after a 12 h fast using the Lysun Blood Lipid Analyzer. Dietary intake was assessed pre‐ and on‐trail using a validated multiple‐pass 24 h recall. Student's paired *t*‐tests evaluated metabolic changes, and regression analyses assessed associations between lipid changes and dietary factors. No lipid exhibited a statistically significant change at the α = 0.05 threshold. However, LDL‐C decreased by 17 mg/dL (*P* = 0.066), suggestive of a biologically meaningful reduction, given the small sample size. Among 81 dietary variables, LDL‐C reduction was significantly associated with decreased intake of added sugars (*P* = 0.030) and ultra‐processed foods (*P* = 0.039) and with increased intake of minimally processed foods (*P* = 0.044), vitamin C (*P* = 0.048) and vitamin K (*P* = 0.047) during hiking. These findings suggest that long‐distance hiking might be associated with lower LDL‐C and that diet quality, particularly food processing level, might be correlated with this trend. This study is the first to link dietary shifts systematically to metabolic outcomes in thru‐hiking, providing hypothesis‐generating insights into the physiological adaptations to prolonged physical exertion and the potential for dietary modulation of lipid metabolism.

## Abstract (highlights)

- **What is the central question of this study?**
  What metabolic outcomes [specifically, changes in total cholesterol, high‐ and low‐density lipoprotein cholesterol (LDL‐C) and triglycerides] are associated with completing the Colorado Trail, and how are pre‐ and on‐trail diet quality measures correlated with these outcomes?
- **What is the main finding and its importance?**
  Thru‐hiking might be associated with reductions in LDL‐C, helping to reconcile conflicting findings in previous case reports. Shifts in diet quality, particularly reductions in added sugars and ultra‐processed foods, are correlated with improvements in LDL‐C. This study is the first to evaluate metabolic outcomes of thru‐hiking beyond individual case reports and to assess the relationship between diet and these outcomes systematically, providing meaningful insights.

## 1 INTRODUCTION

The Colorado Trail (CT) is a 782 km long‐distance hiking trail that spans from the cities of Denver to Durango in the USA, traversing a diverse array of landscapes including high alpine regions, subalpine forests and rugged mountain terrain. CT hikers are individuals who commit to completing the entire trail in a single season (i.e. thru‐hiking), typically between early July and mid‐August (Colorado Trail Foundation, 2024), often taking hikers upwards of a month to complete. This endeavour requires substantial physical endurance, because hikers often cover daily distances exceeding 32 km while managing steep inclines and unpredictable weather conditions (Cole & Thomsen, 2021). Furthermore, thru‐hikers face the challenge of managing their food supplies and hydration, with few opportunities to resupply (Heinbockel & Craighead, 2021). The rigorous demands of long‐distance thru‐hiking present significant physiological stress, with the potential to affect metabolic outcomes. It remains unclear whether the metabolic effects of thru‐hiking are as beneficial as those observed with other forms of sustained physical activity (Twohig‐Bennett & Jones, 2018; Warburton et al., 2006). This warrants further investigation, because existing studies on thru‐hikers report conflicting results. A case study of a Pacific Crest Trail hiker showed increased total cholesterol (TC), triglycerides (TGL) and low‐density lipoprotein cholesterol (LDL‐C), with minimal changes in high‐density lipoprotein cholesterol (HDL‐C) and a marked decrease of bone mineral density (Weiss et al., 2023). Another case study of a Pacific Crest Trail hiker found no changes in body composition and adverse cardiometabolic outcomes (Heinbockel & Craighead, 2021). In contrast, a case study of an Appalachian Trail hiker showed only improved metabolic outcomes, with reductions in TC, TGL and LDL‐C and an increase in HDL‐C (Devoe et al., 2009). These discrepancies highlight the variability of metabolic responses to thru‐hiking and underscore the need for further research beyond individual case studies to better clarify health outcomes associated with thru‐hiking.

To date, no study has examined systematically the relationships between pre‐trail or on‐trail diet and metabolic outcomes in thru‐hikers, highlighting a significant gap in the literature regarding how diet might be associated with health outcomes in this unique population.

In this study, we aimed to address existing discrepancies and gaps by investigating metabolic and nutritional outcomes of individuals who completed the CT. This study focused on changes in blood lipid profiles, specifically of TC, HDL‐C, LDL‐C and TGL from pre‐trail to post‐trail. Blood lipid analysis is a validated method for assessing metabolic changes (Expert Panel on the Detection, Evaluation & Treatment of High Blood Cholesterol in Adults [NCEP], 2001), making it an effective tool for examining metabolic associations of thru‐hiking the CT. We also assessed how both pre‐trail and on‐trail dietary factors were associated with these metabolic outcomes.

Based on some thru‐hiking case reports and general trends associated with physical activity (Devoe et al., 2009; Twohig‐Bennett & Jones, 2018; Warburton et al., 2006), it was hypothesized that thru‐hiking the CT would be associated with an increase in HDL‐C, a decrease in LDL‐C, a decrease in TC and a decrease in TGLs. Given that it is well established that diets high in ultra‐processed foods (UPFs) and added sugars negatively impact blood lipids profiles (Donat‐Vargas et al., 2021; Nouri et al., 2023; Silva Meneguelli et al., 2022; Zhang et al., 2015), it was also hypothesized that an on‐trail diet high in UPFs and added sugars would be associated with attenuated improvements in the lipid profile.

## 2 MATERIALS AND METHODS

### 2.1 Ethical approval

This study was approved by the University of Minnesota Institutional Review Board (IRB no. STUDY00021673) and conducted in accordance with the *Declaration of Helsinki* (2024). The study was registered with ClinicalTrials.gov (NCT06358274) and carried out under Protocol Type HRP‐590 Medical, with CTSI number 32848 and OnCore number MED‐2024‐32848. The principal investigator was Dr Stephen Selinsky, Department of Medicine, Division of Hospital Medicine, University of Minnesota Medical School. All participants provided IRB‐approved written informed consent for participation and publication. Data supporting the findings of this study, including raw data, are available in Supplemental Data Table 1 and Supplemental Data Table 2.

### 2.2 Participants

Participants were recruited via advertisements on CT Foundation online pages, email newsletters and long‐distance hiking websites, targeting individuals planning to hike the CT from early July to mid–late August 2024. Eligibility required participants to be 18–60 years old and with no prior long‐distance hiking experience, defined as having not hiked ≥24 km/day for ≥5 consecutive days, in the past 6 months. Hikers taking statins were excluded. Hikers who disclosed chronic conditions, including hyperlipidaemia, diabetes, liver disease, heart failure and coronary artery disease, were excluded.

### 2.3 Baseline assessments

Prior to the CT hike, participants underwent a baseline assessment within 72 h of their hiking start date, including a 12‐h‐fasted blood lipid panel to measure the concentrations of TC, HDL‐C, LDL‐C and TGL using the Lysun Blood Lipid Analysis Instrument. Samples were obtained from a peripheral vein. Lipid analyses were run in duplicate from the same sample, with two consecutive runs. Duplicate readings were averaged to yield a single reported value. TC, TGL and HDL‐C were measured directly, and LDL‐C was calculated using the Friedewald equation [LDL‐C = TC − HDL‐C − (TG/5)]. The instrument was calibrated with a Certified Control Check before each use. Additional baseline measurements included height, weight, calculated body mass index (BMI) and resting blood pressure (BP). BP was measured on the right arm using a validated automated BP monitor (Omron HEM‐907XL) after 10 min of seated rest. The device automatically recorded three consecutive readings at intervals of 1 min and displayed the average. The Omron HEM‐907XL has been validated against brachial sphygmomanometry and implements the triplicate protocol used in the SPRINT trial (Omboni et al., 2015; SPRINT Research Group, 2015). Participants also completed a pre‐trail survey on demographics, previous long‐distance hiking experience, training and supplement use.

### 2.4 Trail monitoring and post‐trail assessments

During the on‐trail period, participants maintained regular communication with the research team, providing updates on their progress. Upon completion of the CT, participants underwent a post‐trail fasted blood lipid panel assessment using the same instrument between 24 and 72 h after trail completion. Post‐trail lipids were run in duplicate from the same sample. The baseline measurements of height, weight, BMI and BP were repeated to capture any physiological changes resulting from the hike. A post‐trail survey was also administered assessing trail performance metrics (hiking speed, daily mileage, rest days, days to complete).

### 2.5 Dietary data collection and analysis

Dietary intake data were collected using the multiple‐pass 24 h recall method, a validated measure to assess diet (ASA24) (Osadchiy et al., 2020). Pre‐trail dietary habits were documented during the pre‐trail assessment, and on‐trail dietary intake was recorded after the post‐trail assessment. Participants were asked to recall the last full day that they were on trail and were required to finish the recall ≤1 week after their completion date. The ASA24 software was used to analyse both pre‐trail and on‐trail diets, focusing on several key metrics: total caloric intake, macronutrient distribution (carbohydrates, fats and protein), vitamin intake (A–K), sodium, added sugars and alcohol consumption. The software also assessed food categories including whole grains, refined grains, fruits, dark green vegetables, dark orange and red vegetables, legumes, beans, meat, eggs and nuts. Dietary patterns were also analysed using the NOVA food‐processing classification system, which categorizes foods into groups 1 [unprocessed (UPFs) or minimally processed foods (MPFs)], 2 (processed culinary ingredients), 3 (processed foods) and 4 (UPFs and drink products). Caloric intake was assessed as the percentage of total calories from each NOVA group, allowing evaluation of dietary quality and its potential impact on metabolic health. Comparisons between pre‐trail and on‐trail diets were made to assess changes in nutrient intake and food group consumption, with caloric intake also analysed by NOVA group to evaluate shifts in food processing levels.

### 2.6 Statistical analysis

Key demographic variables and metabolic measures (e.g., TC, TGL, HDL‐C, LDL‐C and BP) were assessed for normality using the Shapiro–Wilk test. If variables violated the assumption of normality, non‐parametric methods, specifically the Wilcoxon signed‐rank test, were planned to be used. Data were summarized using proportions for categorical data and descriptive statistics, including the mean and SD. Potential outliers were assessed using a robust outlier detection test (ROUT) with a 1% false‐discovery rate. Student's two‐tailed, paired *t*‐tests were used to assess the significance of metabolic changes on the trail, with change defined as the post‐trail value minus the pre‐trail value. The *P*‐values for all metabolic *t*‐tests were reported, with *P <* 0.05 considered statistically significant. To complement *P*‐values, effect sizes were calculated using Cohen's *d* to quantify effect sizes for the changes in blood lipids.

Results suggesting meaningful changes in blood lipid measures underwent further linear regression analysis to assess the association of the change in blood lipids with several factors, including dietary factors (pre‐trail absolute values, on‐trail values, and changes calculated as on‐trail minus pre‐trail), change in systolic BP (SBP), change in diastolic BP (DBP), absolute weight change, BMI change and hiking performance metrics (training and on‐trail metrics). The *P*‐values for significant blood lipid changes were considered statistically significant at *P <* 0.05.

All significant regression analyses report coefficients, *R*, *R* ^2^ and β values.

## 3 RESULTS

A total of 15 participants enrolled in this study, with three participants terminating the hike early owing to unforeseen circumstances. All three participants who terminated early completed <75% of the trail. These participants were similar in baseline characteristics (mean age = 32 years, mean BMI = 23.6 kg/m^2^; two males, one female) to those who finished, indicating no systematic difference. Analysis, discussion and presentation of results includes the 12 participants who finished the CT. Table 1 presents an overview of group and individual participant demographic information, pre‐trail metabolic characteristics (height, weight, BMI, TC, TGL, HDL‐C, LDL‐C and BP) and responses from the pre‐trail survey.

**TABLE 1:** Participant demographics and pre‐trail characteristics.

Participant	Age (years)	Sex	Height (cm)	Weight (kg)	BMI (kg/m^2^)	TC (mg/dL)	TGL (mg/dL)	HDL‐C (mg/dL)	LDL‐C (mg/dL)	SBP (mmHg)	DBP (mmHg)	Statin use	Supplements	Previous thru‐hike	Distance hiked per week (km)	Times hiked per week (*n*)
1	39	F	162.6	68.2	25.8	175	132	60	89	151	81	No	–	No	24	1
2	29	F	160	65.8	25.7	193^*^	79	54	123^*^	132	85	No	–	Yes	32	2
3	22	M	188	74.8	21.2	157	123	67^*^	65	121	66	No	–	No	19	1
4	22	M	182.9	86.2	25.8	228^*^	201^*^	33^*^	154^*^	124	75	No	–	No	29	1
5	25	M	180.3	77.1	23.7	155	114	68^*^	64	132	87	No	Multivitamin, fish oil	No	23	1
6	31	M	175.3	65.8	21.5	107	63	54	40	122	81	No	–	No	16	1
7	18	M	180.3	72.6	22.4	121	45	55	57	107	70	No	–	No	26	1
8	18	M	167.6	61.2	21.7	121	45	34^*^	78	106	68	No	–	No	21	1
9	23	F	180.3	70.3	21.6	116	65	44^*^	59	112	69	No	Multivitamin	No	18	1
10	39	M	182.9	118	35.3	162	190^*^	44	80	160	92	No	–	No	35	2
11	53	F	170.2	67.1	23.2	206^*^	59	63^*^	131^*^	127	88	No	–	Yes	29	1
12	23	M	185.4	99.8	29	118	51	47	61	129	89	No	–	No	27	1
Mean	28.5	4 F, 8 M	176.3	77.2	24.7	155	97.3	51.9	83.4	127	79.3	12 No 0 Yes	–	10 No 2 Yes	24.9	1.2
95% CI	(22.6, 34.4)	–	(171, 181.5)	(67.9, 86.6)	(22.4, 27.1)	(129.7, 180)	(61.9, 132)	(44.0, 59.5)	(60.9, 105.9)	(116.7, 137.1)	(73, 84.4)	–	–	–	(21.6, 28.2)	(0.9, 1.4)

*Note*: *n* = 12. Abbreviations and terms: BMI, body mass index; CI, confidence interval; DBP, diastolic blood pressure; Distance hiked per week (km), average distance hiked by participant per week in month preceding trail start (from pre‐trail survey); F, female; M = male; HDL‐C, high‐density lipoprotein; LDL‐C, low‐density lipoprotein; Previous thru‐hike, previous thru‐hikes completed by participant anytime within their lifetime (but not within 6 months of the Colorado Trail; from pre‐trail survey); SBP, systolic blood pressure; Statin use, whether the participant is using statins (yes/no; from pre‐trail survey); Supplements, supplements taken by the participant (e.g., multivitamin, iron supplements; from pre‐trail survey); TC, total cholesterol; TGL, triglycerides; Times Hiked per Week, average number of occasions on which participant hiked per week in month preceding trail start (from pre‐trail survey).

* Values outside of clinically normal ranges (TC < 200 mg/dL, TGL < 150 mg/dL, HDL‐C 40——60 mg/dL for males and 50—60 mg/dL for females, LDL‐C < 100 mg/dL).

**FIGURE 1:**
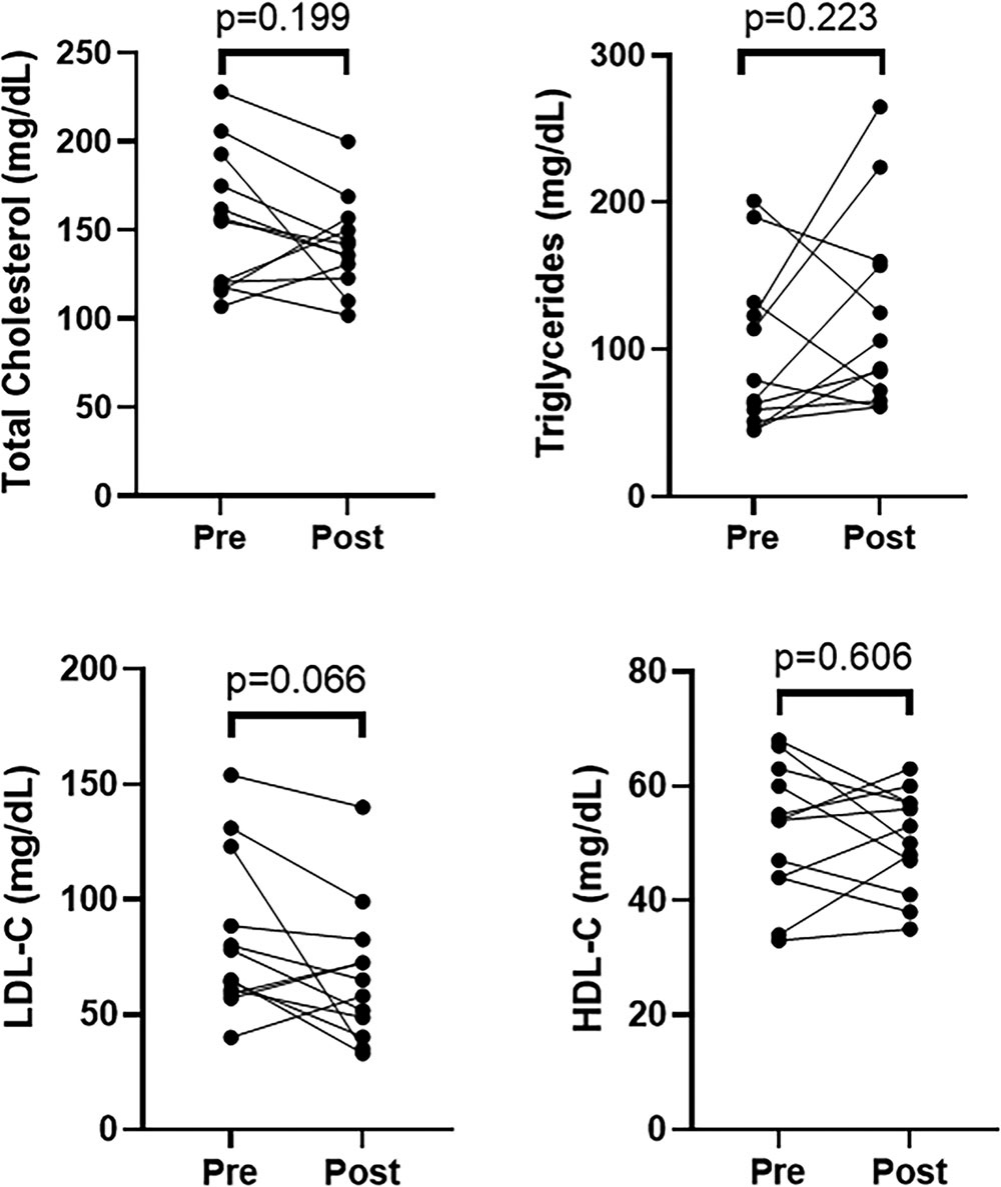
Distribution of pre‐ vs. post‐trail changes for total cholesterol, triglycerides, LDL and HDL. *n* = 12. Abbreviations: HDL‐C, high‐density lipoprotein; LDL‐C, low‐density lipoprotein.

Table 2 presents the group and individual participant post‐trail metabolic characteristics and responses to the post‐trail survey. One participant declined post‐trail BP measurement. Table 3 presents the group and individual participant changes in metabolic characteristics (post minus pre). A negative value indicates a decrease relative to the pre‐trail survey, whereas a positive value indicates an increase. All data were normally distributed. The change in DBP for participant 3 (34 mmHg) was identified as an outlier. This value was excluded from all subsequent analyses. No other data points were detected as outliers. Table 4 presents the results of the Student's paired *t*‐test for metabolic characteristics comparing post‐ with pre‐trail measurements including TC, TGL, HDL‐C, LDL‐C, SBP and DBP. The pre‐ and post‐trail data for the blood lipids are presented in Figure 1. Student's two‐tailed, paired *t*‐tests indicate that no lipid exhibited statistically significant changes at the conventional α = 0.05 threshold (TC, *P* = 0.199; TGL, *P* = 0.223; HDL‐C, *P* = 0.606; and LDL‐C, *P* = 0.066). Given the small sample size (*n* = 12), it is noteworthy that LDL‐C approached the α = 0.05 threshold and remained <0.1. All other lipids had *P *> 0.1. LDL‐C demonstrated a moderate effect size (*d* = 0.52). TC and TGL showed small‐to‐moderate effect sizes (TC, *d* = 0.40; TGL, *d* = −0.34). HDL‐C showed minimal change (*d* = 0.10). Given that the *P*‐value and effect size for LDL‐C were suggestive of a true relationship, it was the sole variable included in the regression analysis for correlation with dietary and other covariate factors (BP, weight loss, BMI, training, and on‐trail hiking metrics).Weight loss (*P* = 2.34 × 10^−5^) and BMI reduction (*P* = 8.33 × 10^−6^) demonstrated statistically significant changes. The investigation of weight and BMI changes and their potential correlation with dietary intake was not a primary focus nor part of the original hypothesis of the study, hence these variables were not included in the dietary correlation analyses. Instead, these variables were analysed in relationship to LDL‐C reduction.

**TABLE 2:** Participant characteristics and cardiometabolic outcomes after completion of the Colorado Trail.

Participant	Weight (kg)	BMI (kg/m^2^)	TC (mg/dL)	TGL (mg/dL)	HDL‐C (mg/dL)	LDL‐C (mg/dL)	SBP (mmHg)	DBP (mmHg)	Hiking speed (km/h)	Average daily distance (km/day)	Rest days (*n*)	Days to complete (*n*)
1	65.8	24.9	144	72	47^*^	83	144	77	2.5	22.5	5	36
2	61.2	23.9	110	61	63	35	–	–	3.9	27.4	0	30
3	63.5	17.6	136	265^*^	50	33	149	100	2.3	27.4	2	30
4	78.9	23.7	200^*^	125	35^*^	140^*^	124	68	3.2	25.8	2	31
5	72.1	22.2	142	224^*^	57	40	108	88	2.6	25.8	2	31
6	58.5	19	131	85	56	58	130	100	3.2	30	1	27
7	62.1	19.1	150	87	60	73	111	72	3.8	32.2	2	25
8	55.8	19.8	123	106	48	52	123	69	4.3	32.2	7	25
9	61.2	18.8	157	157^*^	53	72	114	80	4	30	1	27
10	102.1	30.6	135	160^*^	38^*^	65	135	84	2.5	24.2	3	33
11	59	20.4	169	65	57	99	119	83	2.7	24.2	2	33
12	83.9	24.2	102	61	41	49	128	80	2.6	24.2	4	33
Mean	68.6	22.1	141.7	122.3	50.4	66.6	125.9	81.9	3.10	28.8	2.58	30
95% CI	(61, 76.3)	(19.9, 24.1)	(125, 158)	(76.7, 167.9)	(45.4, 55.4)	(49.6, 83.5)	(118.5, 133.3)	(75.7, 88.1)	(2.7, 88.1)	(26.2, 31.4)	(1.5, 3.6)	(27.8, 32.2)

*Note*: Abbreviations and terms: BMI, body mass index; CI, confidence interval; DBP, diastolic blood pressure; HDL‐C, high‐density lipoprotein; LDL‐C, low‐density lipoprotein; SBP, systolic blood pressure; TC, total cholesterol; TGL, triglycerides.

* Values outside of clinically normal ranges (TC < 200 mg/dL, TGL < 150 mg/dL, HDL‐C 40–60 mg/dL for males and 50–60 mg/dL for females, LDL‐C < 100 mg/dL).

**TABLE 3:** Change in characteristics.

Participant	Weight (kg)	BMI (kg/m^2^)	TC (mg/dL)	TGL (mg/dL)	HDL‐C (mg/dL)	LDL‐C (mg/dL)	SBP (mmHg)	DBP (mmHg)
1	−2.4	−0.9	−31	−60	−13	−6	−7	−4
2	−4.6	−1.8	−83	−18	9	−88	–	–
3	−11.3	−3.6	−21	142	−17	−32	28	–
4	−7.3	−2.1	−28	−76	2	−14	0	−7
5	−5	−1.5	−13	110	−11	−24	−24	1
6	−7.3	−2.5	24	22	2	18	8	19
7	−10.5	−3.3	29	42	5	16	4	2
8	−5.4	−1.9	2	61	14	−26	17	1
9	−9.1	−2.8	41	92	9	13	2	11
10	−15.9	−4.7	−27	−30	−6	−15	−25	−8
11	−8.1	−2.8	−37	6	−6	−32	−8	−5
12	−15.9	−4.8	−16	10	−6	−12	−1	−9
Mean	−8.57	−2.73	−13.3	25.1	−1.5	−16.8	−0.545	3.18
95% CI	(−11.3, −5.9)	(−3.5, −2)	(−34.8, 8.2)	(−18, 68)	(−7.7, 4.7)	(−35, 1.3)	(−11.1, 10)	(−5.6, 12.2)

*Note*: *n* = 12. All ‘change’ characteristics were calculated as follows: (value post‐trail) − (value pre‐trail ) = (change in value). Abbreviations: BMI, body mass index; CI, confidence interval; DBP, diastolic blood pressure; HDL‐C, high‐density lipoprotein; LDL‐C, low‐density lipoprotein; SBP, systolic blood pressure; TC, total cholesterol; TGL, triglycerides.

**TABLE 4:** Summary of lipid and blood pressure changes.

Characteristic	Mean	95% CI	SD	*P*‐value
Weight^*^	−8.567	(−11.269, −5.864)	4.253	2.34 × 10^−5^
BMI^*^	−2.725	(−3.494, −1.956)	1.211	8.33 × 10^−6^
TC	−13.333	(−34.809, 8.142)	33.8	0.199
TGL	25.083	(−17.703, 67.87)	67.341	0.223
HDL‐C	−1.5	(−7.711, 4.711)	9.775	0.606
LDL‐C	−16.833	(−35.001, 1.334)	28.594	0.066
SBP	−0.545	(−11.101, 10.010)	15.712	0.911
DBP	3.182	(−5.750, 12.114)	13.295	0.446

*Note*: *n* = 12. Abbreviations: BMI, body mass index; CI, confidence interval; DBP, diastolic blood pressure; HDL‐C, high‐density lipoprotein; LDL‐C, low‐density lipoprotein; SBP, systolic blood pressure; TC, total cholesterol; TGL, triglycerides.

^*^ *P <* 0.05.

Group and individual participant dietary data for pre‐trail, on‐trail, and the change from pre‐trail to on‐trail in all measured dietary factors are presented in Table S1. The *P*‐values and *R*‐values for measured dietary factors associated with LDL‐C reduction in the statistical regression analysis are presented in Table S2. Statistically significant factors include change in added sugar (*P* = 0.0298), change in vitamin C (*P* = 0.0480), change in vitamin K (*P* = 0.0470), change in percentage of calories from UPFs (*P* = 0.0391) and change in percentage of calories from MPFs (*P* = 0.0442). No other dietary factors were found to be significantly correlated with LDL‐C reduction.

Table 5 presents additional regression statistics for the dietary factors significantly correlated with LDL‐C reductions, including *R*, *R* ^2^ and β values, to provide further context on the nature of these correlations. Regression analysis revealed that added sugars exhibited the strongest negative correlation with LDL‐C reduction (*R* ^2^ = 0.425), followed by UPFs (*R* ^2^ = 0.393). MPFs (*R* ^2^ = 0.377), vitamin C (*R* ^2^ = 0.368) and vitamin K (*R* ^2^ = 0.359) demonstrated positive correlations with LDL‐C reduction.

**TABLE 5:** Regression analysis of significant dietary factors associated with low‐density lipoprotein reduction.

Variable	Multiple *R*	*R* ^2^	β	*P*‐value
ΔMPFs	0.614	0.377	−0.03	0.0442
ΔUPFs	0.627	0.393	0.09	0.0391
ΔVit K	0.599	0.359	−134	0.0470
ΔVit C	0.607	0.368	−0.15	0.0480
ΔAdded Sugars	0.652	0.425	17	0.0298

*Note*: *n* = 12. All Δ characteristics were calculated as follows: (value post‐trail) − (value pre‐trail ) = (change in value). Abbreviations: ΔAdded Sugars, change in grams of added sugar; ΔMPFs, change in percentage of calories from minimally processed foods; ΔUPFs, change in percentage of calories from ultra‐processed foods; ΔVitC, change in micrograms of vitamin C; ΔVitK, change in micrograms of vitamin K.

Regression analyses also assessed the associations between changes in LDL‐C and changes in body weight, BMI, SBP and DBP. There were no significant associations between the reduction in LDL‐C and any of these variables (body weight, *P* = 0.308; BMI, *P* = 0.398; SBP, *P* = 0.714; and DBP, *P* = 0.748).

Table 6 presents the *P*‐values for the association between hiking characteristics, including training metrics, on‐trail hiking speed, on‐trail average daily distance, on‐trail rest days, and days to complete, and LDL‐C reduction, as assessed through regression analysis. None of these variables was significantly associated with LDL‐C reduction.

**TABLE 6:** *P*‐values for the association between hiking characteristics and low‐density lipoprotein reduction.

Characteristic	Multiple *R*	*R* ^2^	β	*P*‐value
Training distance hiked per week (km)	0.48	0.23	−2.32	0.114
Training times hiked per week (*n*)	0.56	0.32	−41.62	0.212
On‐trail hiking speed (km/h)	0.02	0.01	−0.50	0.967
On‐trail average daily mileage (km/day)	0.25	0.06	2.19	0.427
On‐trail rest days (*n*)	0.26	0.07	−1.3	0.410
Days to complete (*n*)	0.23	0.06	−1.9	0.461

*Note*: Terminology: Training distance hiked per week, average distance hiked by participant per week in month preceding trail start (from pre‐trail survey); Training times hiked per week, average occasions on which participant hikes per week in month preceding trail start (from pre‐trail survey); On‐trail hiking speed, average daily hiking speed in kilometres per hour on‐trail (from post‐trail survey); On‐trail average daily mileage, average kilometres hiked per day on‐trail (from post‐trail survey); On‐trail rest days, days over the course of the entire trail when participant reported having hiked 0 miles (from post‐trail survey); Days to complete, total number of days spent hiking the entire trail not including rest days (from post‐trail survey).

## 4 DISCUSSION

The primary aim of this study was to investigate the metabolic and nutritional outcomes of individuals who completed the CT. This included examining changes in TC, HDL‐C, LDL‐C and TGL, in addition to the relationships between dietary factors and lipid changes from pre‐trail to on‐trail.

Results indicated that no lipid exhibited statistically significant changes at the α = 0.05 threshold. However, LDL‐C demonstrated a reduction of 17 mg/dL with a *P*‐value of 0.066, suggestive of a true relationship given our sample size. This interpretation is supported further by effect size measurements (e.g., Cohen's *d*), which indicate a meaningful magnitude of change. This suggests that it is worthwhile to investigate LDL‐C reduction further to see whether more data can confirm the meaningful reduction found in this study. Moreover, the aim of the present study was to investigate metabolic outcomes of completing the CT; it would be reductionist not to highlight these results as a possible biologically meaningful outcome in the context of a small sample size.

In contrast, all other lipids had *P*‐values > 0.1, suggesting a minimally meaningful trend, even in the setting of a small sample size. Their confidence intervals broadly spanned zero, further indicating that changes in these lipids had limited association. TC and TGL showed small to moderate effect sizes, and HDL‐C changed minimally, suggesting a lack of biologically meaningful change.

The suggested LDL‐C reduction aligns with evidence that regular physical activity lowers LDL‐C, probably through established physiological mechanisms (Stanton et al., 2022). Physical activity has been shown to decrease degradation of LDL‐C receptors via modulation of proprotein convertase subtilisin/kexin type 9 (PCSK9) and sterol regulatory element‐binding protein (SREBP) gene activity, thereby enhancing the clearance of LDL‐C from the bloodstream (Tirandi et al., 2022; Wen et al., 2013). It is plausible that thru‐hiking exerts similar effects on these pathways.

The lack of meaningful changes in TC, TGL and HDL‐C contrasts with established exercise literature, which typically reports decreased TC and TGL and increased HDL‐C (NCEP, 2001; Stanton et al., 2022). Similar deviations have been observed in other thru‐hiking contexts, such as a case study of a Pacific Crest Trail hiker that reported increased TC and TGL with minimal change in HDL‐C; however, that report also observed an increase in LDL‐C. These atypical responses might result from physiological adaptations to prolonged low energy availability, including hypothalamic–pituitary axis suppression and hormonal shifts that reduce cholesterol utilization for steroidogenesis and impair lipid metabolism (Weiss et al., 2023). It remains mechanistically unclear why LDL‐C responded in line with typical physical activity adaptations, whereas TC, TGL and HDL‐C did not. These findings highlight the complex physiological demands of thru‐hiking and underscore the need for further studies to characterize driving mechanisms better.

Given that LDL‐C was the only lipid with suggestion of a meaningful change, it was the only parameter included in the regression analysis for dietary and hiking performance metrics. In this study, we investigated the regression correlation of 81 dietary factors with LDL‐C reduction. Change in added sugars exhibited the strongest negative correlation with LDL‐C reduction, followed by change in UPFs. Change in MPFs, change in vitamin C and change in vitamin K demonstrated significant positive correlation with LDL‐C reduction. No other dietary factors were significantly related to the reduction in LDL‐C. These results differ slightly from the original hypothesis that on‐trail added sugars and UPFs would be associated with attenuated lipid improvements. Rather, the greater association appears to be for the change from pre‐trail to on‐trail diet, rather than solely the on‐trail diet. As hypothesized and as supported by literature (Donat‐Vargas et al., 2021; Nouri et al., 2023; Silva Meneguelli et al., 2022; Zhang et al., 2015), increased consumption of UPFs and added sugar are associated with lesser reductions in LDL‐C levels.

With findings indicating significance in the change in added sugars, UPFs, MPFs, vitamin C and vitamin K only, results suggest that metabolic outcomes during thru‐hiking might be influenced more by the overall dietary shift towards or away from processed, sugar‐rich or fresh foods than by absolute on‐trail nutrient intake.

These observed dietary associations might be attributed to established metabolic effects of dietary composition and processing. Processed foods are typically higher in added sugars, which contribute to adverse lipid profiles via mechanisms such as increased insulin resistance, hepatic lipogenesis and elevated very low‐density lipoprotein production (Babalola et al., 2025). Additionally, food processing might impair lipid metabolism through increased generation of reactive oxygen species, with less fresh and more processed foods promoting oxidative stress and lipid deterioration (Juul et al., 2021; Maldonado‐Pereira et al., 2023). However, the metabolic impact of food processing remains an active area of research, and most studies focus on static dietary patterns rather than dynamic shifts in food quality. Findings from the present study might reflect metabolic stresses and adaptations triggered by dietary transitions during prolonged physical exertion, highlighting a potentially underexplored link between change in food quality and lipid metabolism.

All participants experienced weight and BMI reduction, indicating an on‐trail calorie deficit. These findings align with previous studies, which have consistently reported thru‐hiking being associated with weight loss (Devoe et al., 2009; Heinbockel & Craighead, 2021; Weiss et al., 2023). Given that weight loss and BMI reduction were not the metabolic outcomes of interest in the present study nor part of the hypothesis, they were not correlated with diet. Instead, weight loss and reductions in BMI were assessed in relationship to LDL‐C to explore their significance in metabolic outcomes. There were no relationships between changes in weight and BMI and changes in LDL‐C, suggesting that weight loss itself might not be the primary driver of improved LDL‐C. Likewise, the reduction in LDL‐C was not correlated with SBP or DBP. These findings might help to rule out weight loss or BP as confounders, reinforcing that diet might be a key factor influencing LDL‐C changes.

The study assessed hiking training metrics, including weekly distance and hiking frequency in the month before the trail, neither of which showed a significant correlation with LDL‐C reductions. On‐trail factors, such as hiking speed, average daily distance, rest days, and days to complete the trail were not correlated with LDL‐C changes. These findings suggest that diet might have a stronger impact on changes in LDL‐C with thru‐hiking than training or performance‐related factors.

Clinical interpretation of LDL‐C reduction was evaluated. The 17 mg/dL reduction in LDL‐C observed in this study might be clinically meaningful, because such a reduction would be expected to reduce cardiovascular event risk by ∼10% (Mhaimeed et al., 2024). Additionally, three participants exhibited clinically elevated LDL‐C pre‐trail, and two of these participants saw their LDL‐C levels return to clinically normal ranges post‐trail. Although the third participant did not return to a clinically normal range, their LDL‐C dropped from 154 pre‐trail to 140 post‐trail. No participant who started with normal LDL‐C levels pre‐trail experienced an increase in LDL‐C that exceeded clinically normal ranges. These findings highlight that clinically meaningful changes in LDL‐C were observed in the majority of participants with clinical LDL‐C concerns, reiterating that thru‐hiking might be associated with meaningful LDL‐C reduction. It is important to note that the aim of the present study was not to evaluate the clinical utility of recommending thru‐hiking as a formal intervention for lipid improvement; instead, our goal was to provide hikers with information about expected changes in blood lipids during a thru‐hike.

It is important to note that there was notable individual variability in the responses of some subjects regarding LDL‐C and SBP. In Table 2, subjects 2 and 3 had low LDL‐C levels of 35 and 33 mg/dL, respectively. In Table 3, subject 2 showed an 88 mg/dL decrease in LDL‐C. None of these values was classified as an outlier, but they lack explanation and might affect the overall mean LDL‐C results. Likewise, SBP changes in subjects 3, 5 and 10 (28, −24 and −25 mmHg) were not outliers but lack explanation and might influence the mean BP findings.

### 4.1 Experimental considerations

This study had several strengths and limitations. This study represents the largest known investigation of metabolic outcomes in long‐distance hikers to date, with previous studies being limited to smaller case reports; even so, 12 participants is a small sample size. The small number of participants limited our power to detect statistically significant outcomes. The study instead focused on effect size and further investigated relationships with *P <* 0.1. The convenience sampling method might also have limited our statistical power. Multiple linear regression models were not pursued owing to the limited sample size, to avoid overfitting and unstable estimates. Future studies should aim to include a greater number of participants.

The cohort (mean age 28.5 years, 33% female, and 83% first‐time hikers) was moderately younger and included more first‐time hikers than a 2024 Pacific Crest Trail hiker demographic survey (mean age 38 years, 40% female, and 50% first‐time hikers), although the sex distribution was similar (Fox, 2024). These similarities suggest that our findings have moderate generalizability to the broader thru‐hiking population, but might be especially applicable to novice hikers. However, the convenience sample and the lack of explicit screening for disorders affecting fat absorption or lipoprotein metabolism might limit generalizability.

This single‐arm study lacked a randomized control group, introducing the possibility that unmeasured confounding variables, such as individual food budgets, might have influenced dietary intake and lipid outcomes. The lack of a control group also hindered the ability to establish causal relationships between dietary changes and metabolic outcomes.

An important consideration for this study is whether the variability in lipids and BP observed pre to post thru‐hiking is physiological or methodological. LDL‐C was not measured directly in this study but estimated using the Friedewald formula, which is sensitive to elevated TGL and might underestimate LDL‐C in such cases, potentially affecting result accuracy. Furthermore, the Lysun blood analyser, although CE‐certified and manufactured under ISO 13485 standards, has limited peer‐reviewed data available regarding its precision and clinical performance. In the present study, all duplicate lipid analyses differed by ≤3 mg/dL, supporting the internal reliability of the device. Nevertheless, the variability inherent to estimated LDL‐C and the limited external validation of the analyser for all lipid values is an important consideration and potential limitation of this study. Future studies would benefit from using instrumentation with established validation and direct LDL‐C measurement to strengthen the reliability and generalizability of lipid measurements.

BP was measured in triplicate with the Omron HEM‐907XL automated device. This device has been validated extensively against brachial sphygmomanometry and demonstrates high reliability and reproducibility (Omboni et al., 2015). In addition, the same triplicate protocol was used in the landmark SPRINT trial (SPRINT Research Group, 2015). A limitation is that only the average of three measurements was recorded. However, data from our laboratory in six individuals using the same device in triplicate mode demonstrated minimal intra‐measurement variation (mean difference, ±1.3 mmHg SBP and ±1.1 mmHg DBP). Variation between averages was also within ±2 mmHg for both SBP and DBP for repeat triplicate measurements, supporting the internal reliability and reproducibility of the device. Overall, the use of a well‐validated monitor suggests that thru‐hiking is likely to be associated with inconsistent changes in BP. Future studies could explore mechanisms underlying the wide variability in BP responses to thru‐hiking.

Post‐trail blood samples were collected 24–72 h after completion. Although this reduces the likelihood of capturing only acute effects from the final day, participants sampled closer to 24 h might still have been more affected. More standardized timing would improve comparability in future studies.

The study assessed overall weight change but did not differentiate between fat mass and lean mass loss, a limitation with important implications for interpreting the health effects of the weight reduction. Future studies should assess body composition to clarify the health impacts.

The ASA24 is likely to have provided a reasonable estimate of on‐trail intake, because literature shows that multi‐day backpackers and athletes often consume highly repetitive meals (Sundqvist, 2023). Nonetheless, a single 24 h recall might not have captured the entire trail diet fully, which could impact dietary results. Future studies could explore methods to assess dietary intake across the full trail period.

Perhaps the most important consideration of this study is the potential utility of its findings: that LDL‐C reduction might be enhanced by consuming equal or greater amounts of fresh, unprocessed foods while thru‐hiking compared with pre‐trail. However, the logistics, demands and cost of maintaining such a diet in backcountry settings might severely limit its feasibility for thru‐hikers. Future research is needed to explore practical strategies for incorporating these dietary improvements into hiking routines while navigating the constraints of extended outdoor expeditions.

Future research should focus on randomized clinical trials with larger samples of thru‐hikers to test controlled dietary interventions (UPFs vs. MPFs) and further elucidate the impact of food processing on metabolic health within this population. Such studies would provide more robust evidence on how diet influences performance and health outcomes. Future investigations should explore the delineation of metabolic outcomes specific to thru‐hiking or endurance exercise more broadly, particularly where inconsistencies in findings exist.

## 5 CONCLUSION

This study examined metabolic outcomes associated with thru‐hiking and found that completing a thru‐hike might be associated with a reduction in LDL‐C, although this relationship did not reach statistical significance. Minimally meaningful trends were observed for TC, TGL or HDL‐C, indicating that thru‐hiking is not associated with changes in these blood lipids.

Significant correlations between LDL‐C reduction and changes in added sugars, UPFs, MPFs and vitamins C and K suggest that relative shifts in dietary quality, rather than absolute on‐trail intake, might be most relevant to lipid changes. These findings support the consideration of food processing and freshness as potentially modifiable factors that influence metabolic outcomes during extended physical activity.

Overall, this study contributes to the literature by systematically evaluating relationships between thru‐hiking, lipid outcomes and dietary changes. LDL‐C decreased in 9 of 12 participants, suggesting that thru‐hiking might generally be associated with reduced LDL‐C. However, owing to the small sample size and lack of statistical significance, these findings should be considered hypothesis generating rather than definitive conclusions. Furthermore, the identification of specific dietary shifts associated with LDL‐C changes might provide insight into how diet quality might influence metabolic outcomes during prolonged physical exertion, warranting exploration in larger studies. These findings have implications for both the thru‐hiking community and the broader field of metabolic health research.

## AUTHOR CONTRIBUTIONS

All data were collected in the field in northern Colorado. Kiaya Johnston: Conceptualization, Data Curation, Formal Analysis, Investigation, Methodology, Writing—Original Draft, Writing—Review & Editing. Stephen Selinsky: Conceptualization, Methodology. Benjamin Langworthy: Methodology, Formal Analysis, Writing—Review & Editing. Daniel Craighead: Conceptualization, Methodology, Formal Analysis, Supervision, Writing—Review & Editing. All authors have approved the final version of the manuscript and agree to be accountable for all aspects of the work in ensuring that questions related to the accuracy or integrity of any part of the work are appropriately investigated and resolved. All persons designated as authors qualify for authorship, and all those who qualify for authorship are listed.

## CONFLICT OF INTEREST

None declared.

## Supporting information




Supplemental Data Table 1: Individual Diet Data (Pre‐Trail, On‐Trail, and Pre‐Trail vs On Trail) Supplemental Data Table 2: P‐Values of Dietary Factors Correlated with Change in LDL‐C

## Data Availability

No additional data from this study will be made publicly available.

## References

[eph70175-bib-0001] Babalola, O. O. , Akinnusi, E. , Ottu, P. O. , Bridget, K. , Oyubu, G. , Ajiboye, S. A. , Waheed, S. A. , Collette, A. C. , Adebimpe, H. O. , Nwokafor, C. V. , Oni, E. A. , Aturamu, P. O. , & Iwaloye, O. (2025). The impact of ultra‐processed foods on cardiovascular diseases and cancer: Epidemiological and mechanistic insights. Advanced Molecular Medicine, 5, 100072.

[eph70175-bib-0002] Cole, T. , & Thomsen, J. M. (2021). Navigating the challenges of the multi‐phase thru‐hiking experience. Journal of Outdoor Recreation, Education, and Leadership, 13(3), 52–69.

[eph70175-bib-0003] Colorado Trail Foundation . (2024). The Colorado Trail Foundation . https://coloradotrail.org

[eph70175-bib-0004] Devoe, D. , Israel, R. G. , Lipsey, T. , & Voyles, W. (2009). A long‐duration (118‐day) backpacking trip (2669 km) normalizes lipids without medication: A case study. Wilderness & Environmental Medicine, 20(4), 347–352. 20030443 10.1580/1080-6032-020.004.0347

[eph70175-bib-0005] Donat‐Vargas, C. , Sandoval‐Insausti, H. , Rey‐García, J. , Moreno‐Franco, B. , Åkesson, A. , Banegas, J. R. , Rodríguez‐Artalejo, F. , & Guallar‐Castillón, P. (2021). High consumption of ultra‐processed food is associated with incident dyslipidemia: A prospective study of older adults. Journal of Nutrition, 151(8), 2390–2398. 34038538 10.1093/jn/nxab118

[eph70175-bib-0006] Expert Panel on the Detection, Evaluation, and Treatment of High Blood Cholesterol in Adults . (2001). Executive summary of the third report of the National Cholesterol Education Program (NCEP) Expert Panel on Detection, Evaluation, and Treatment of High Blood Cholesterol in Adults (Adult Treatment Panel III). The Journal of the American Medical Association, 285(19), 2486–2497. 11368702 10.1001/jama.285.19.2486

[eph70175-bib-0007] Fox, T. (2024). Pacific Crest Trail Hiker Survey (2024). Halfway Anywhere. https://www.halfwayanywhere.com/trails/pacific‐crest‐trail/pct‐hiker‐survey‐2024/

[eph70175-bib-0008] Heinbockel, T. C. , & Craighead, D. H. (2021). Impact of a long‐distance hike on the Pacific Crest Trail on arterial function and body composition in a highly fit young male. Physiological Reports, 9(5), e14767. 33661563 10.14814/phy2.14767 PMC7931801

[eph70175-bib-0009] Juul, F. , Vaidean, G. , & Parekh, N. (2021). Ultra‐processed foods and cardiovascular diseases: Potential mechanisms of action. Advances in Nutrition, 12(5), 1673–1680. 33942057 10.1093/advances/nmab049 PMC8483964

[eph70175-bib-0010] Maldonado‐Pereira, L. , Barnaba, C. , & Medina‐Meza, I. G. (2023). Oxidative status of ultra‐processed foods in the Western diet. Nutrients, 15(23), 4873. 38068731 10.3390/nu15234873 PMC10708126

[eph70175-bib-0011] Mhaimeed, O. , Burney, Z. A. , Schott, S. L. , Kohli, P. , Marvel, F. A. , & Martin, S. S. (2024). The importance of LDL‐C lowering in atherosclerotic cardiovascular disease prevention: Lower for longer is better. American Journal of Preventive Cardiology, 18, 100649. 38576462 10.1016/j.ajpc.2024.100649 PMC10992711

[eph70175-bib-0012] Nouri, M. , Eskandarzadeh, S. , Makhtoomi, M. , Rajabzadeh‐Dehkordi, M. , Omidbeigi, N. , Najafi, M. , & Faghih, S. (2023). Association between ultra‐processed foods intake with lipid profile: A cross‐sectional study. Scientific Reports, 13(1), 7258. 37142735 10.1038/s41598-023-34451-x PMC10160124

[eph70175-bib-0013] Omboni, S. , Parati, G. , & Mancia, G. (2015). Validation of the omron HEM‐907 oscillometric blood pressure monitor according to the european society of hypertension international protocol. Blood Pressure Monitoring, 12(4), 233–242. 10.1097/MBP.0b013e32813fa386 17625396

[eph70175-bib-0014] Osadchiy, T. , Poliakov, I. , Olivier, P. , Rowland, M. , & Foster, E. (2020). Progressive 24‐hour recall: Usability study of short retention intervals in web‐based dietary assessment surveys. Journal of Medical Internet Research, 22(2), e13266. 32012055 10.2196/13266 PMC7055775

[eph70175-bib-0015] Silva Meneguelli, T. , Juvanhol, L. L. , da Silva Leite, A. , Bressan, J. , & Hermsdorff, H. H. M. (2022). Minimally processed versus processed and ultra‐processed food in individuals at cardiometabolic risk. British Food Journal, 124(3), 811–832.

[eph70175-bib-0016] SPRINT Research Group . (2015). A randomized trial of intensive versus standard blood‐pressure control. New England Journal of Medicine, 373(22), 2103–2116. 26551272 10.1056/NEJMoa1511939 PMC4689591

[eph70175-bib-0017] Stanton, K. M. , Kienzle, V. , Dinnes, D. L. M. , Kotchetkov, I. , Jessup, W. , Kritharides, L. , Celermajer, D. S. , & Rye, K. A. (2022). Moderate‐ and high‐intensity exercise improves lipoprotein profile and cholesterol efflux capacity in healthy young men. Journal of the American Heart Association, 11(12), e023386. 35699182 10.1161/JAHA.121.023386 PMC9238648

[eph70175-bib-0018] Thompson, P. D. , Lazarus, B. , Cullinane, E. , Henderson, L. O. , Musliner, T. , Eshleman, R. , & Herbert, P. N. (1983). Exercise, diet, or physical characteristics as determinants of HDL levels in endurance athletes. Journal of Clinical Pharmacology, 23(4), 263–267. 10.1016/0021-9150(83)90182-x 6405759

[eph70175-bib-0019] Tirandi, A. , Montecucco, F. , & Liberale, L. (2022). Physical activity to reduce PCSK9 levels. Frontiers in Cardiovascular Medicine, 9, 988698. 36093150 10.3389/fcvm.2022.988698 PMC9453490

[eph70175-bib-0020] Twohig‐Bennett, C. , & Jones, A. (2018). The health benefits of the great outdoors: A systematic review and meta‐analysis of greenspace exposure and health outcomes. Environmental Research, 166, 628–637. 29982151 10.1016/j.envres.2018.06.030 PMC6562165

[eph70175-bib-0021] Warburton, D. E. , Nicol, C. W. , & Bredin, S. S. (2006). Health benefits of physical activity: The evidence. Canadian Medical Association Journal, 174(6), 801–809. 16534088 10.1503/cmaj.051351 PMC1402378

[eph70175-bib-0022] Weiss, E. P. , Frech, A. M. , & Perez, V. R. (2023). Low energy availability and increased risk of relative energy deficiency in sport (RED‐S) during a 3767‐km thru‐hike on the Pacific Crest Trail: A case study. Wilderness & Environmental Medicine, 34(4), 536–542. 37586947 10.1016/j.wem.2023.06.011

[eph70175-bib-0023] Wen, S. , Jadhav, K. S. , Williamson, D. L. , & Rideout, T. C. (2013). Treadmill exercise training modulates hepatic cholesterol metabolism and circulating PCSK9 concentration in high‐fat‐fed mice. Journal of Lipids, 2013, 1–9. 10.1155/2013/908048 PMC3703876 23862065

[eph70175-bib-0024] Zhang, Z. , Gillespie, C. , Welsh, J. A. , Hu, F. B. , & Yang, Q. (2015). Usual intake of added sugars and lipid profiles among U.S. adolescents: National Health and Nutrition Examination Survey, 2005–2010. Journal of Adolescent Health, 56(3), 352–359. 10.1016/j.jadohealth.2014.12.001 PMC4494648 25703323

