# Supplementary material for: Lipid profiles and nutritional dynamics of long‐distance hiking: A longitudinal study on the Colorado Trail
Source: Exp Physiol. 2025 Dec 28;111(4):1960–70. doi: 10.1113/EP093118 (PMC13140263; doi:10.1113/EP093118)
Supplement: Supplementary file 1 — Supplemental Data Table 1: Individual Diet Data (Pre‐Trail, On‐Trail, and Pre‐Trail vs On Trail) Supplemental Data Table 2: P‐Values of Dietary Factors Correlated with Change in LDL‐C [file EPH-111-1960-s001.docx]

Supplemental Data Table 1: Individual Diet Data (Pre-Trail, On-Trail, and Pre-Trail vs On-Trail)

| Dietary Characteristic | Participant 1 | Participant 2 | Participant 3 | Participant 4 | Participant 5 | Participant 6 | Participant 7 | Participant 8 | Participant 9 | Participant 10 | Participant 11 | Participant 12 |
| --- | --- | --- | --- | --- | --- | --- | --- | --- | --- | --- | --- | --- |
| Pre-trail Calories (kcal) | 1394 | 1967 | 4853 | 2821 | 1983 | 1759 | 1866 | 2085 | 1822 | 2570 | 1295 | 2257 |
| Pre-trail Carbs as % of Total Calories (%) | 40 | 49 | 36 | 45 | 66 | 49 | 51 | 58 | 42 | 55 | 34 | 42 |
| Pre-trail Fat as % of Total Calories (%) | 41 | 32 | 33 | 36 | 13 | 32 | 31 | 25 | 35 | 28 | 30 | 42 |
| Pre-trail Protein as % of Total Calories (%) | 19 | 18 | 11 | 16 | 11 | 19 | 18 | 17 | 23 | 17 | 35 | 16 |
| Pre-trail Alcohol as % of Total Calories (%) | 0 | 0 | 20 | 3 | 10 | 0 | 0 | 0 | 0 | 0 | 0 | 0 |
| Pre-trail Whole Grains (oz) | 0.7 | 0.7 | 0 | 1.3 | 1.6 | 3.2 | 2.6 | 4.7 | 2.4 | 1.3 | 0.7 | 6.6 |
| Pre-trail Refined Grains (oz) | 0 | 1.7 | 21.9 | 9.8 | 7.5 | 1.5 | 5.1 | 4.8 | 0 | 9.3 | 0.4 | 3.7 |
| Pre-trail Fruit (cups) | 4 | 1.2 | 0 | 0.8 | 1.8 | 2 | 1.5 | 2.5 | 5.4 | 1.8 | 3.2 | 0 |
| Pre-trail Dark Green Vegetables (cups) | 0.7 | 0 | 0 | 0 | 0.1 | 1.3 | 0.5 | 0.3 | 2.1 | 0 | 1.1 | 0.1 |
| Pre-trail Dark Red and Orange Vegetables (cups) | 0.8 | 0.3 | 0.6 | 0.3 | 1.9 | 1.4 | 0 | 0.3 | 0.9 | 0.1 | 0.4 | 1 |
| Pre-trail Legumes and Beans (cups) | 0 | 0 | 0 | 0 | 0 | 0.1 | 0 | 0 | 0 | 0 | 0 | 0 |
| Pre-trail Meat, Poultry, and Eggs (oz) | 3.8 | 8 | 5.6 | 0.2 | 0 | 3.2 | 7 | 7 | 10 | 6.8 | 7.5 | 6.2 |
| Pre-trail Nuts (oz) | 4.3 | 0 | 0.8 | 1.5 | 2.8 | 7.3 | 0.5 | 0.7 | 3.7 | 0 | 2.3 | 0.2 |
| Pre-trail Added Sugar (g) | 30 | 131 | 20 | 102 | 120 | 28 | 20 | 119 | 1 | 90 | 18 | 120 |
| Pre-trail Saturated Fats (g) | 10 | 35 | 57 | 56 | 7 | 16 | 16 | 48 | 17 | 25 | 7 | 39 |
| Pre-trail Sodium (mg) | 2085 | 2856 | 5964 | 4126 | 2622 | 2625 | 2809 | 2683 | 2318 | 5115 | 1821 | 3827 |
| Pre-trail Carbohydrates (g) | 143 | 244 | 440 | 315 | 233 | 221 | 234 | 301 | 194 | 353 | 112 | 240 |
| Pre-trail Protein (g) | 69 | 14 | 135 | 110 | 55 | 85 | 88 | 90 | 106 | 111 | 116 | 90 |
| Pre-trail Vitamin A (mcg) | 1117 | 724 | 977 | 1000 | 1167 | 207 | 420 | 526 | 1679 | 357 | 657 | 1354 |
| Pre-trail Vitamin B6 (mg) | 1.4 | 2.8 | 3.6 | 2.2 | 2 | 2.4 | 4.6 | 2.8 | 2.6 | 3 | 2.4 | 1.9 |
| Pre-trail Vitamin B12 (mcg) | 2 | 6 | 7 | 4 | 3 | 3 | 8 | 8 | 2 | 6 | 3 | 8 |
| Pre-trail Vitamin C (mg) | 168 | 63 | 17 | 57 | 176 | 113 | 114 | 104 | 251 | 59 | 109 | 25 |
| Pre-trail Vitamin D (IU) | 50 | 323 | 169 | 52 | 4 | 0 | 306 | 53 | 168 | 260 | 0 | 304 |
| Pre-trail Vitamin E (mg) | 10 | 13 | 15 | 16 | 18 | 19 | 13 | 11 | 21 | 10 | 18 | 8 |
| Pre-trail Vitamin K (mcg) | 226 | 19 | 118 | 35 | 96 | 209 | 268 | 67 | 380 | 115 | 333 | 40 |
| Pre-trail % Calories from Ultraprocessed Foods (%) | 11% | 74% | 92.4% | 93.9% | 81% | 48.4% | 11% | 69.9% | 15% | 83% | 29% | 84% |
| Pre-trail % Calories from Minimally Processed Foods (%) | 63% | 15% | 6% | 5% | 15% | 42% | 60% | 20% | 75% | 10% | 57% | 8% |
|  |  |  |  |  |  |  |  |  |  |  |  |  |
| On-trail Calories (kcal) | 2974 | 3266 | 4041 | 2284 | 3219 | 4308 | 3263 | 3257 | 3604 | 1978 | 1759 | 2829 |
| On-trail Carbs as % of Total Calories (%) | 55 | 56 | 62 | 33 | 63 | 47 | 65 | 50 | 59 | 55 | 34 | 60 |
| On-trail Fat as % of Total Calories (%) | 33 | 34 | 42 | 25 | 28 | 39 | 24 | 40 | 42 | 28 | 52 | 27 |
| On-trail Protein as % of Total Calories (%) | 12 | 10 | 12 | 13 | 7 | 14 | 12 | 10 | 15 | 17 | 13 | 13 |
| On-trail Alcohol as % of Total Calories (%) | 0 | 0 | 2 | 0 | 2 | 0 | 0 | 0 | 3 | 0 | 0 | 0 |
| On-trail Whole Grains (oz) | 1.5 | 3.3 | 1.8 | 1.7 | 1.6 | 1.1 | 6.5 | 2.5 | 6.4 | 2.2 | 2.1 | 3.3 |
| On-trail Refined Grains (oz) | 4.9 | 3.5 | 10 | 6.3 | 12.7 | 7.8 | 9.8 | 4.5 | 2.3 | 2.8 | 4.1 | 1.7 |
| On-trail Fruit (cups) | 2.7 | 0 | 0.1 | 0.4 | 0.6 | 0.3 | 0.3 | 0 | 1.1 | 1 | 0.5 | 3.1 |
| On-trail Dark Green Vegetables (cups) | 0 | 0 | 0.3 | 0 | 0 | 0 | 0 | 0 | 0 | 0 | 0 | 0 |
| On-trail Dark Red and Orange Vegetables (cups) | 0.6 | 0 | 0.3 | 0 | 1 | 0.1 | 0 | 0 | 0 | 0 | 0 | 0 |
| On-trail Legumes and Beans (cups) | 0.6 | 0.2 | 0.2 | 0.1 | 0.4 | 0.2 | 0.2 | 0.1 | 0.1 | 0 | 0.3 | 0.3 |
| On-trail Meat, Poultry, and Eggs (oz) | 0 | 0.9 | 2 | 0 | 0 | 1.1 | 0 | 0.2 | 0.7 | 5.6 | 2.5 | 3.9 |
| On-trail Nuts (oz) | 13.7 | 5.1 | 20.7 | 4.3 | 3.3 | 6.1 | 3.9 | 6.7 | 6.5 | 0.3 | 4.7 | 1.7 |
| On-trail Added Sugar (g) | 47 | 162 | 53 | 129 | 147 | 229 | 191 | 122 | 94 | 98 | 29 | 155 |
| On-trail Saturated Fats (g) | 19 | 35 | 40 | 19 | 23 | 75 | 23 | 56 | 74 | 18 | 29 | 32 |
| On-trail Sodium (mg) | 2757 | 3858 | 2979 | 2338 | 2831 | 2909 | 3627 | 3343 | 5261 | 2534 | 2449 | 4091 |
| On-trail Carbohydrates (g) | 428 | 467 | 450 | 366 | 526 | 519 | 532 | 415 | 368 | 227 | 154 | 436 |
| On-trail Protein (g) | 95 | 79 | 130 | 75 | 56 | 148 | 96 | 84 | 150 | 84 | 59 | 93 |
| On-trail Vitamin A (mcg) | 443 | 856 | 615 | 147 | 314 | 524 | 2074 | 675 | 1221 | 283 | 150 | 316 |
| On-trail Vitamin B6 (mg) | 2.3 | 4 | 4 | 2.3 | 2 | 3.9 | 6.3 | 2.5 | 4.3 | 1.8 | 0.9 | 24.5 |
| On-trail Vitamin B12 (mcg) | 3 | 8 | 5 | 6 | 2.4 | 10 | 11 | 6 | 14 | 2 | 2 | 25 |
| On-trail Vitamin C (mg) | 95 | 332 | 229 | 63 | 119 | 134 | 120 | 148 | 169 | 129 | 11 | 158 |
| On-trail Vitamin D (IU) | 41 | 147 | 0 | 322 | 1 | 39 | 190 | 104 | 247 | 127 | 26 | 58 |
| On-trail Vitamin E (mg) | 21 | 14 | 52 | 8 | 21 | 25 | 21 | 11 | 30 | 9 | 18 | 7 |
| On-trail Vitamin K (mcg) | 92 | 112 | 157 | 45 | 117 | 82 | 68 | 92 | 72 | 85 | 65 | 59 |
| On-trail % Calories from Ultraprocessed Foods (%) | 20% | 61% | 68% | 85% | 48% | 87.40% | 88% | 74% | 89% | 79% | 40.30% | 50.80% |
| On-trail % Calories from Minimally Processed Foods (%) | 60% | 25% | 15% | 12% | 36% | 8% | 6% | 24% | 4% | 15% | 50% | 41% |
|  |  |  |  |  |  |  |  |  |  |  |  |  |
| Δ Calories (kcal) | 1580 | 1299 | -812 | -537 | 1236 | 2549 | 1397 | 1172 | 1782 | -592 | 464 | 572 |
| Δ Carbs as % of Total Calories (%) | 15 | 7 | 6 | 18 | -3 | -2 | 14 | -8 | -3 | 0 | 0 | 18 |
| Δ Fat as % of Total Calories (%) | -8 | 2 | 9 | -11 | 15 | 7 | -7 | 15 | 7 | 0 | 22 | -15 |
| Δ Protein as % of Total Calories (%) | -7 | -8 | 1 | -3 | -4 | -5 | -6 | -7 | -8 | 0 | -22 | -3 |
| Δ Alcohol as % of Total Calories (%) | 0 | 0 | -18 | -3 | -8 | 0 | 0 | 0 | 3 | 0 | 0 | 0 |
| Δ Whole Grains (oz) | 4.3 | 2.6 | 1.8 | 0.4 | 0 | -2.1 | 3.9 | -2.2 | 4 | 0.9 | 1.4 | -3.3 |
| Δ Refined Grains (oz) | 4.9 | 1.8 | -11.9 | -3.5 | 5.2 | 6.3 | 4.7 | -0.3 | 2.3 | -6.5 | 3.7 | -2 |
| Δ Fruit (cups) | -1.3 | -1.2 | 0.1 | -0.4 | -1.2 | -1.7 | -1.2 | -2.5 | -4.3 | -0.8 | -2.7 | 3.1 |
| Δ Dark Green Vegetables (cups) | -0.7 | 0 | 0.3 | 0 | -0.1 | -1.3 | -0.5 | -0.3 | -2.1 | 0 | -1.1 | -0.1 |
| Δ Dark Red and Orange Vegetables (cups) | -0.2 | -0.3 | -0.3 | -0.3 | -0.9 | -1.3 | 0 | -0.3 | -0.9 | -0.1 | -0.4 | -1 |
| Δ Legumes and Beans (cups) | 0.6 | 0.2 | 0.2 | 0.1 | 0.4 | 0.1 | 0.2 | 0.1 | 0.1 | 0 | 0.3 | 0.3 |
| Δ Meat, Poultry, and Eggs (oz) | -3.8 | -7.1 | -3.6 | -0.2 | 0 | -2.1 | -7 | -6.8 | -9.3 | -1.2 | -5 | -2.3 |
| Δ Nuts (oz) | 9.4 | 5.1 | 19.9 | 2.8 | 0.5 | -1.2 | 3.4 | 6 | 2.8 | 0.3 | 2.4 | 1.5 |
| Δ Added Sugar (g) | 17 | 31 | 33 | 27 | 27 | 201 | 171 | 3 | 93 | 8 | 11 | 35 |
| Δ Saturated Fats (g) | 9 | 0 | -17 | -37 | 16 | 59 | 7 | 8 | 57 | -7 | 22 | -7 |
| Δ Sodium (mg) | 672 | 1002 | -2985 | -1788 | 209 | 284 | 818 | 660 | 2943 | -2581 | 628 | 264 |
| Δ Carbohydrates (g) | 285 | 223 | 10 | 51 | 293 | 298 | 298 | 114 | 174 | -126 | 42 | 196 |
| Δ Protein (g) | 26 | 65 | -5 | -35 | 1 | 63 | 8 | -6 | 44 | -27 | -57 | 3 |
| Δ Vitamin A (mcg) | -674 | 132 | -362 | -853 | -853 | 317 | 1654 | 149 | -458 | -74 | -507 | -1038 |
| Δ Vitamin B6 (mg) | 0.9 | 1.2 | 0.4 | 0.1 | 0 | 1.5 | 1.7 | -0.3 | 1.7 | -1.2 | -1.5 | 22.6 |
| Δ Vitamin B12 (mcg) | 1 | 2 | -2 | 2 | -0.6 | 7 | 3 | -2 | 12 | -4 | -1 | 17 |
| Δ Vitamin C (mg) | -73 | 269 | 212 | 6 | -57 | 21 | 6 | 44 | -82 | 70 | -98 | 133 |
| Δ Vitamin D (IU) | -9 | -176 | -169 | 270 | -3 | 39 | -116 | 51 | 79 | -133 | 26 | -246 |
| Δ Vitamin E (mg) | 11 | 21 | 37 | 2 | 13 | 6 | 8 | 0 | 9 | -1 | 0 | -1 |
| Δ Vitamin K (mcg) | -134 | 93 | 39 | 10 | 21 | -127 | -200 | 25 | -308 | -30 | -268 | 19 |
| Δ % Calories from Ultraprocessed Foods (%) | 9% | -13% | -24% | -9% | -33% | 39% | 77% | 4% | 74% | -4% | 11% | -33% |
| Δ % Calories from Minimally Processed Foods (%) | -3% | 10% | 9% | 7% | 21% | -34% | -54% | 4% | -71% | 5% | -7% | 33% |

n=12

All units are noted in diet category field

Δ values were calculated by: Δ=(post-trail)-(pre-trail)

Supplemental Data Table 2: P-Values of Dietary Factor Correlated with ΔLDL-C

| **Dietary Factor Correlated with ΔLDL-C** | **P-value** | **Multiple R-value** |
| --- | --- | --- |
| Pre-trail Calories (kcal) | 0.657 | 0.15 |
| Pre-trail Carbs as % of Total Calories (%) | 0.977 | 0.01 |
| Pre-trail Fat as % of Total Calories (%) | 0.677 | 0.14 |
| Pre-trail Protein as % of Total Calories (%) | 0.904 | 0.04 |
| Pre-trail Alcohol as % of Total Calories (%) | 0.600 | 0.17 |
| Pre-trail Whole Grains (oz) | 0.263 | 0.37 |
| Pre-trail Refined Grains (oz) | 0.698 | 0.11 |
| On-trail Fruit (cups) | 0.365 | 0.31 |
| Pre-trail Dark Green Vegetables (cups) | 0.093 | 0.53 |
| Pre-trail Dark Red and Orange Vegetables (cups) | 0.542 | 0.21 |
| Pre-trail Legumes and Beans (cups) | 0.224 | 0.39 |
| Pre-trail Meat, Poultry, and Eggs (oz) | 0.778 | 0.09 |
| Pre-trail Nuts (oz) | 0.107 | 0.51 |
| Pre-trail Added Sugar (g) | 0.058 | 0.58 |
| Pre-trail Saturated Fats (g) | 0.368 | 0.29 |
| Pre-trail Sodium (mg) | 0.774 | 0.09 |
| Pre-trail Carbohydrates (g) | 0.691 | 0.14 |
| Pre-trail Protein (g) | 0.109 | 0.5 |
| Pre-trail Vitamin A (mcg) | 0.970 | 0.01 |
| Pre-trail Vitamin B6 (mg) | 0.681 | 0.14 |
| Pre-trail Vitamin B12 (mcg) | 0.572 | 0.19 |
| Pre-trail Vitamin C (mg) | 0.222 | 0.39 |
| Pre-trail Vitamin D (IU) | 0.581 | 0.19 |
| Pre-trail Vitamin E (mg) | 0.099 | 0.07 |
| Pre-trail Vitamin K (mcg) | 0.082 | 0.58 |
| Pre-trail % Calories from Ultraprocessed Foods (%) | 0.142 | 0.47 |
| Pre-trail % Calories from Minimally Processed Foods (%) | 0.113 | 0.49 |
|  |  |  |
| On-trail Calories (kcal) | 0.539 | 0.2 |
| On-trail Carbs as % of Total Calories (%) | 0.163 | 0.07 |
| On-trail Fat as % of Total Calories (%) | 0.674 | 0.14 |
| On-trail Protein as % of Total Calories (%) | 0.161 | 0.45 |
| On-trail Alcohol as % of Total Calories (%) | 0.633 | 0.16 |
| On-trail Whole Grains (oz) | 0.349 | 0.31 |
| On-trail Refined Grains (oz) | 0.664 | 0.14 |
| On-trail Fruit (cups) | 0.461 | 0.25 |
| On-trail Dark Green Vegetables (cups) | 0.643 | 0.16 |
| On-trail Dark Red and Orange Vegetables (cups) | 0.821 | 0.07 |
| On-trail Legumes and Beans (cups) | 0.642 | 0.15 |
| On-trail Meat, Poultry, and Eggs (oz) | 0.871 | 0.05 |
| On-trail Nuts (oz) | 0.689 | 0.13 |
| On-trail Added Sugar (g) | 0.571 | 0.19 |
| On-trail Saturated Fats (g) | 0.323 | 0.33 |
| On-trail Sodium (mg) | 0.670 | 0.14 |
| On-trail Carbohydrates (g) | 0.372 | 0.12 |
| On-trail Protein (g) | 0.107 | 0.51 |
| On-trail Vitamin A (mcg) | 0.368 | 0.3 |
| On-trail Vitamin B6 (mg) | 0.711 | 0.13 |
| On-trail Vitamin B12 (mcg) | 0.363 | 0.3 |
| On-trail Vitamin C (mg) | 0.091 | 0.52 |
| On-trail Vitamin D (IU) | 0.582 | 0.18 |
| On-trail Vitamin E (mg) | 0.928 | 0.03 |
| On-trail Vitamin K (mcg) | 0.161 | 0.45 |
| On-trail % Calories from Ultraprocessed Foods (%) | 0.072 | 0.56 |
| On-trail % Calories from Minimally Processed Foods (%) | 0.127 | 0.47 |
|  |  |  |
| Δ Calories (kcal) | 0.403 | 0.28 |
| Δ Carbs as % of Total Calories (%) | 0.982 | 0.01 |
| Δ Fat as % of Total Calories (%) | 0.548 | 0.2 |
| Δ Protein as % of Total Calories (%) | 0.642 | 0.15 |
| Δ Alcohol as % of Total Calories (%) | 0.510 | 0.23 |
| Δ Whole Grains (oz) | 0.850 | 0.06 |
| Δ Refined Grains (oz) | 0.504 | 0.22 |
| Δ Fruit (cups) | 0.699 | 0.13 |
| Δ Dark Green Vegetables (cups) | 0.092 | 0.53 |
| Δ Dark Red and Orange Vegetables (cups) | 0.302 | 0.34 |
| Δ Legumes and Beans (cups) | 0.436 | 0.25 |
| Δ Meat, Poultry, and Eggs (oz) | 0.854 | 0.06 |
| Δ Nuts (oz) | 0.334 | 0.32 |
| Δ Added Sugar (g) | 0.0298 | 0.65 |
| Δ Saturated Fats (g) | 0.181 | 0.43 |
| Δ Sodium (mg) | 0.673 | 0.14 |
| Δ Carbohydrates (g) | 0.559 | 0.19 |
| Δ Protein (g) | 0.980 | 0.01 |
| Δ Vitamin A (mcg) | 0.513 | 0.22 |
| Δ Vitamin B6 (mg) | 0.744 | 0.11 |
| Δ Vitamin B12 (mcg) | 0.242 | 0.38 |
| Δ Vitamin C (mg) | 0.0480 | 0.61 |
| Δ Vitamin D (IU) | 0.367 | 0.3 |
| Δ Vitamin E (mg) | 0.251 | 0.38 |
| Δ Vitamin K (mcg) | 0.0470 | 0.6 |
| Δ % Calories from Ultraprocessed Foods (%) | 0.0391 | 0.63 |
| Δ % Calories from Minimally Processed Foods (%) | 0.0442 | 0.61 |
